# Fission yeast Ase1^PRC1^ is required for the G_2_-microtubule damage response

**DOI:** 10.22099/mbrc.2021.41001.1650

**Published:** 2021-12

**Authors:** Rose M. Doss, Sindi Xhunga, Dorothy Klimczak, Molly Cameron, Jordan Verlare, Tom D. Wolkow

**Affiliations:** Department of Biology, University of Colorado at Colorado Springs, Colorado Springs, CO 80918

**Keywords:** G_2_-microtubule damage checkpoint Rad26^ATRIP^ Ase1^PRC1^

## Abstract

*Schizosaccharomyces pombe* delays entry into mitosis following G_2_ microtubule damage. This pathway is dependent on Rad26^ATRIP^, the regulatory subunit of the Rad26^ATRIP^/Rad3^ATR^ DNA damage response (DDR) complex. However, this G_2_ microtubule damage response pathway acts independently of the G_2_ DNA damage checkpoint pathway. To identify other proteins in this G_2_ microtubule damage pathway, we previously screened a cDNA overexpression library for genes that rescued the sensitivity of *rad26Δ* cells to the microtubule poison thiabendazole. A partial cDNA fragment encoding only the C-terminal regulatory region of the microtubule bundling protein *Ase1*^PRC1 ^was isolated. This fragment lacks the Ase1^PRC1^ dimerization and microtubule binding domains and retains the conserved C-terminal unstructured regulatory region. Here, we report that *ase1Δ* cells fail to delay entry into mitosis following G_2_ microtubule damage. Microscopy revealed that Rad26^ATRIP^ foci localized alongside Ase1^PRC1^ filaments, although we suggest that this is related to microtubule-dependent double strand break mobility that facilitates homologous recombination events. Indeed, we report that the DNA repair protein Rad52 co-localizes with Rad26^ATRIP ^at these foci, and that localization of Rad26^ATRIP^ to these foci depends on a Rad26^ATRIP^ N-terminal region containing a checkpoint recruitment domain. To our knowledge, this is the first report implicating Ase1^PRC1^ in regulation of the G_2_/M transition.

## INTRODUCTION

A diverse array of intrinsic and extrinsic insults threatens genomic integrity, and those that lead to double strand DNA breaks (DSBs) are particularly toxic. In humans, repair of DSBs occurs primarily via non-homologous end-joining (NHEJ) and homologous dependent repair (HDR) [1, 2]. NHEJ and HDR are stimulated by a trinity of synergistic phosphatidylinositol 3-kinase-related kinases (PIKKs): DNA-dependent protein kinase catalytic subunit (DNA-PKcs); ataxia-telangiectasia mutated (ATM); and ataxia-telangiectasia and Rad3-related (ATR) that initiate a number of other DDR responses as well, including cell cycle checkpoint control over G_1_/S, G_2_/M and S-phase [3–6]. ATM and ATR have also been implicated in pathways that respond to interphase microtubule (MT) damage. For example, MT toxins cause G_1_ and G_2_ cell cycle arrest in cultured cells by mechanisms that target downstream components of ATM and ATR signaling, including CHK1, CDC25C and CDC2 [7–11]. Exactly why eukaryotic cells keep the integrity of interphase microtubules under surveillance, and how they detect compromised interphase MTs, are areas for exploration. 

Yeast model systems provide a framework for dissecting human DDR pathways [12]. In fission yeast, the conserved Rad26^ATRIP^/Rad3^ATR^ complex directs DDRs similar to those in humans. The Rad26^ATRIP^/Rad3^ATR^ complex also participates in a pathway that delays entry into mitosis following G_2_-MT damage [13]. Rad26^ATRIP^ plays a cytoplasmic role during this response, as disruption of its C-terminal nuclear export signal prevents both cytoplasmic accumulation of Rad26^ATRIP^ and the G_2_/M delay that follows treatment with MT poisons. Notably, this nuclear export allele of *rad26*^ATRIP^ does not compromise the DDR. Therefore, the checkpoint responses to DNA and G_2_-MT damage are genetically separate pathways dependent upon Rad26^ATRIP^. Also using fission yeast, Balestra and Jimenez [14] demonstrated that G_2_-MTdamage delays entry into mitosis through stabilization of Wee1^WEE1^, a negative regulator of mitotic cyclin-dependent kinase (CDK) activity and target of the DDR [15–17]. We therefore suspect that the Rad26^ATRIP^/Rad3^ATR^ complex initiates stabilization of Wee1^WEE1^ when G_2_-MTs are damaged. Here, we present evidence that the microtubule bundling protein Ase1^PRC1^ is another component of this G_2_-MT damage response in fission yeast. 

## MATERIALS AND METHODS

**PCR cloning of** ***ase1*** **and** ***rad26*** **into GFP-tagging expression vectors:** PCR amplification of *ase1 *and *rad26* cDNAs from a cDNA library (gift of M. Yamamoto) were performed using high-fidelity Phusion Master Mix (MO531S; New England Biolabs). Primer sequences appear in Table 1. Amplicons were cloned using the pJET blunt-end cloning kit (K1231; Thermo Scientific), transformed into DH5-Alpha Mix & Go! competent cells (T3007; ZYMO Research), and recombinant vectors were purified using a midi prep kit (12143; QIAGEN). cDNA inserts were then directionally cloned into the thiamine-repressible, amino-terminal pRep41 eGFP and LEU2 vector [18] as NdeI-BamHI fragments and transformed into yeast using the yeast transformation protocol described by Bähler et al [19].

**Table 1:** Primers used in this study

**Primer Set Name**	**Sequence**	**Resulting protein Size, aa**
Full *Ase1* gene	Forward: P-5’ CAT ATG CAA ACA GTA ATG ATG 3’Reverse: P-5’ GGA TCC TTA AAA GCC TTC TTC 3’	731
*Ase1-C-term*	Forward: P-5’ CAT ATG GCT ATG ACG AGT CCA 3’Reverse: P-5’ GGA TCC TTA AAA GCC TTC TTC 3’	218
1 Full* Rad26* gene	Forward: P-5’ CAT ATG ATG ATG GCT GAT GAA AGT 3’ Reverse: P-5’ GGA TCC CTA AAA ATT AGT GTA CAA 3’	615
2 *Rad26Δ*_1–103aa_	Forward: P-5’ CAT ATG TCC GAA GCT AAT AAT GCC 3’ Reverse: P-5’ GGA TCC CTA AAA ATT AGT GTA CAA 3’	512
3 *Rad26Δ*_310–615aa_	Forward: P-5’ AT ATG ATG ATG GCT GAT GAA AGT 3’ Reverse: P-5’ ACA TCA TCG TCG ATT TAG GAT CC 3’	309
4 *Rad26Δ*_109–615aa_	Forward: P-5’ AT ATG ATG ATG GCT GAT GAA AGT 3’ Reverse: P-5’ GGA TCC CTA TGA GGC TTG TGA GTT TAC GG 3’	108
5 *Rad26Δ*_1–25aa_*–Δ*_147–615aa_	Forward: P-5’ CAT ATG GAA TTA GAG CAA CAA GCT CAA ACC 3’ Reverse: P-5’ GGA TCC CTA CTC ATG AAA TAG GGA TTT CGT 3’	121
6 *Rad26Δ*_1–40aa_*–Δ*_147–615aa_	Forward: P-5’ CAT ATG GTA GTT GTA CCG AGT GAA AAG CAA 3’ Reverse: P-5’ GGA TCC CTA CTC ATG AAA TAG GGA TTT CGT 3’	106
7 *Rad26Δ*_113–123aa_	Forward: P-5’ CAA CGG AAA TTA GAG GAG CTT AAA AAA GAA 3’ Reverse: P-5’ TAA CGA TTC TAG GGC GGC ATT ATT AGC TTC 3’	604

**Physiological methods:** Spindle-pole-body (SPB) separation was monitored in different strains containing* cdc25.22* and the SPB marker *cut12-egfp* [20]. Cells of each strain were cultured in YE5S liquid medium to optical density (OD) 0.3 at 30°C, then shifted to 37°C for 3 hours 5 minutes before 16 mg/ml Carbendzim (MBC) was added. Cultures were maintained in the presence of MBC at 37°C for another 25 minutes before downshifting to 20°C and releasing cells from the *cdc25.22* block. The percentage of cells containing two Cut12-EGFP foci was determined every 20 minutes following this downshift. Three trials were performed, and 200 cells were scored at every 20-minute time point. Strains used in this study are outlined in Table 2.

**Table 2:** Fission yeast strains used in this study

**Strain**	**Genotype**	**Origin**
TE236	*leu1-32 ura 4-d18 h−*	Kostrub *et al., *(1998)
TE257	*rad26::ura4+ ade6-704 leu1-32 ura4-D18 h− *	Al-Khodairy *et al.,* (1994)
TW1197	*rad26-GFP::kanR leu1-32 ura 4-d18 h−*	Baschal *et al.,* (2006)
MCI728	*z:adh15:mcherry-atb2:natMX6 leu1-32 ura-D18 h+*	gift of Meredith Betterton
FY20056	*ase1-GFP::kanR leu1 h−*	Yeast Resource Center, yeast.nig.ac.jp
ST754	*ase1D::kanR leu1-32 ura4-D18 h+ *	gift of Thibault Courtheoux
TW1341	*ase1D::kanR cdc25.22 cut12-gfp*	this study
TW1345	*ase1D::kanR rad26::ura+ cdc25.22 cut12-GFP*	this study
TW1300	*rad26::ura4+ cdc25.22 cut12-egfp:ura4+*	Herring *et al.,* (2010)
MKSP765	*rad26-mcherry::kanR leu1-32 ura4-D18 h+ *	gift of Megan King
MKSP2074	*rad52-mcherry::kanr ura4-D18 leu1-32 his3-D1*	gift of Megan King
FY20720	*sad1-mcherry::KanR leu1 his2 h+*	Yeast Resource Center, yeast.nig.ac.jp

**Microscopy:** To visualize EGFP fusion proteins, 1 ml aliquots from cultures grown in liquid YE5S to OD 0.3 were centrifuged and resuspended in cold methanol for one minute, washed twice in 100 ml SlowFade Component C (SlowFade Antifade Kit, Molecular Probes) and air dried on coverglass. Once dried, 4.5 μl SlowFade Component A was dropped on the coverglass, which was then placed onto a slide. Achieving yeast monolayers that adhered tightly to the coverslips was crucial to observing fluorescence signals. To help ensure that such layers formed, the coverglass was soaked in acetone for one day, scrubbed with dishwashing soap, wiped with 70% ethanol and air dried prior to use. Images were acquired using a Leica DM5000 equipped with a Leica DFC350FX R2 digital camera and Leica FW4000 software.

## RESULTS AND DISCUSSION

**Figure 1:**
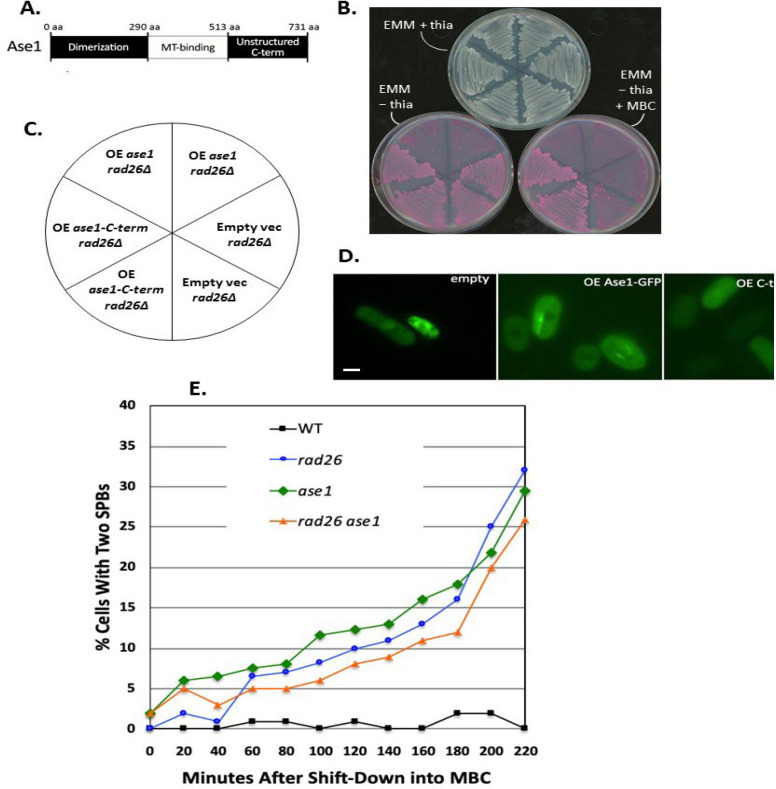
**Ase1 is required to delay SPB separation following G**_2_ **-microtubule damage. A. **Map of the dimerization domain, MT-binding domain and C-terminal region of Ase1.** B. **Overexpression of the C terminal (513–731) amino acids of Ase1 rescues the MBC sensitivity of *rad26Δ* cells. Thiamine (thia) represses transcription of vector inserts. In this experiment, a medium strength *nmt *thiamine-repressible promoter was fused to full length Ase1-GFP, Ase1-C-term-GFP, or empty GFP vector, and transformed into *rad26Δ* cells under repressive conditions (EMM + thiamine). Two transformants representing each condition were streaked to repressive conditions (EMM + thiamine) for two days before replica plating to media with inducing conditions and a pink vital dye. **C. **Map of the genotypic distributions on the EMM-thia replica plates shown in (**B**). **D. **Overexpressed full length Ase1-GFP assembled into bright linear structures within nonviable cells. Overexpressed Ase1-C-term was diffusely localized throughout viable cells. Bar = 5 μm. **E. **Ase1 is required to delay SPB separation following MBC treatment. Strains containing the *cdc25.22* and *cut12-gfp* alleles were arrested in G_2_ before releasing into medium with or without 16 mg/ml MBC. At each time point, the percentage of cells containing two Cut12-GFP foci was calculated from 150 cells. WT refers to *rad26*^+^* ase1*^+^ cells with *cdc25.22 cut12-gfp* alleles in the background

Previously, we screened a cDNA library for genes that, when overexpressed (OE), permit *rad26Δ* cells to grow on medium containing thiabendazole (TBZ) [21]. Of 10,000 transformants, four cDNAs representing Rad24, SPCC70.01, DASH complex subunit Dam1, and the C-terminal 218 amino acids of Ase1 (aa 513–731; Fig. 1A) were identified. Here, we investigate the role of Ase1 in the *rad26*-dependent G_2_-MT damage response more closely, since both *ase1*^+^ and *rad26*^+^ are required for proper cell morphology and minichromosome stability [13, 22]. Ase1 is a conserved MT-bundling protein containing a dimerization domain, a MT-binding domain, and an unstructured C-term [22–26]. Below, we confirm that OE of the unstructured Ase1-C-term rescues the growth of *rad26Δ* cells on media containing a MT toxin, and report that Ase1 is required to delay mitotic entry following insult to G_2_-MTs.

 We observed *nmt*-promoter driven OE of full length Ase1-GFP to be toxic, presumably due to MT-hyper-bundling (Fig. 1B, C; [22]). Within these nonviable cells, we observed that OE Ase1-GFP assembled into bright linear structures (Fig. 1D), consistent with MT-hyper-bundling at overlapping microtubules [22]. Overexpression of the 218aa unstructured C-term of Ase1 was not toxic, and rescued *rad26Δ* growth on carbendazim (MBC; Fig. 1B, C). This C-term fragment localized diffusely throughout cells (Fig. 1D) and did not appear to integrate within the yeast MT cytoskeleton. Therefore, OE Ase1-C-term may rescue *rad26Δ* growth on MBC in a regulatory manner, as opposed to a structural one.

Next, we tested if *ase1*^+^ is required for the *rad26*^+^-dependent checkpoint response to G_2_-MT damage (Fig. 1E). The temperature-sensitive *cdc25.22* allele was used to reversibly block cells in G_2_, and the spindle-pole-body (SPB) marker Cut12-EGFP was used to monitor SPB separation and mitotic entry [20,27,28]. After wildtype cells were released from the G_2_ block into nutrient rich YE5S medium containing MBC, SPB separation was prevented for greater than three hours (Fig. 1E). This result is consistent with those of Akera, Sato and Yamamoto [29], who observed that SPBs fail to separate during MBC treatment. Following release of *rad26Δ, ase1Δ, *and* rad26Δ ase1Δ* cells from the G_2_ block into MBC media, precocious SPB separation began within the first hour in all three strains (Fig. 1E). That the kinetics of SPB separation were similar among the three strains suggests that Ase1 and Rad26 operate in the same pathway. We also tested if OE Ase1-C-term restored the G_2_-delay to *rad26Δ* cells during treatment with MT-toxin. However, the *nmt*-promoter requires growth in nutrient-deficient, synthetic media to drive OE, and we observed that cells grown under these nutrient-limiting conditions fail to delay G_2_/M following treatment with MT poisons (data not shown). This may be due to activation of a stress MAP kinase response that overrides this G_2_-MT damage response in nutrient-depleted conditions [30]. In summary, we conclude that Ase1 participates in the Rad26-dependent G_2_-MT damage response pathway. 

**Figure 2:**
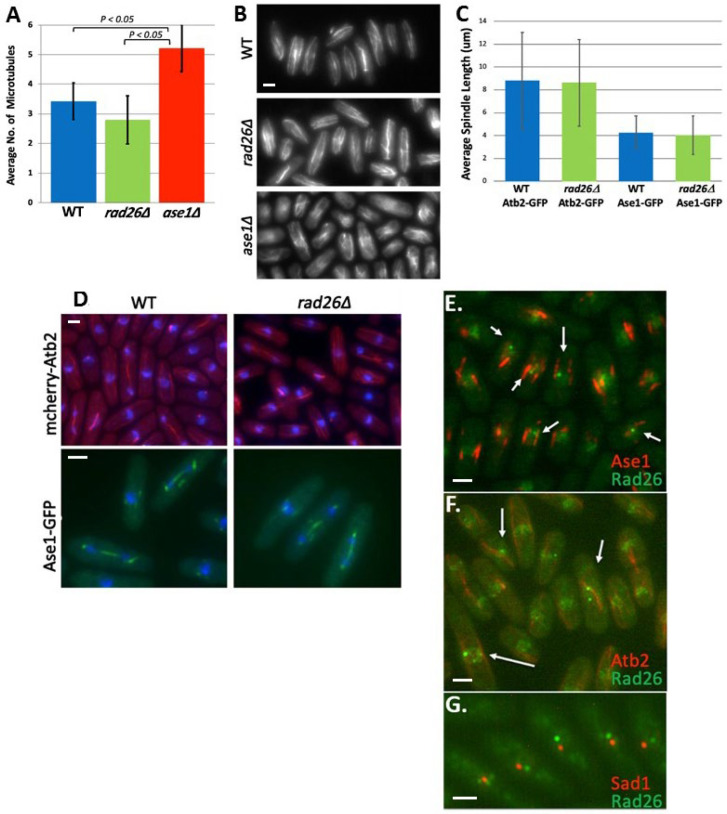
**Rad26 does not influence MT structure, although it does tangentially localize with Ase1 and MTs.** All strains were grown at 30ºC in liquid YE5S to optical density 0.3 and fixed with cold methanol. The number of MT- bundles per cell (**A, B**) and spindle length (**C, D**) were normal in *rad26Δ* cells. (**E, F **and **G**) Rad26 tangentially localizes with Ase1 and MTs, but not the SPB. Strains expressing mcherry-Atb2 (MTs), Rad26-GFP, Rad26-mcherry, Ase1-GFP and Sad1-mcherry were used in these experiments (false colors are sometimes shown); DAPI (blue) was used to visualize nuclei in (**D**). All bars =5 μm

Ase1 bundles MTs, and its loss results in an increased number of interphase MT bundles, as well as short mitotic spindles that are prone to collapse [22,31]. In agreement, we observed that the number of MT bundles in *ase1Δ *cells was significantly greater than the numbers in WT and *rad26Δ *cells, which were similar (Fig. 2A, B). In addition, the length of mitotic spindles decorated with either Atb2-GFP or Ase1-GFP was similar in WT and *rad26Δ* cells (Fig. 2C, D). Therefore, *rad26*^+^ does not appear to share *ase1*^+^-dependent functions related to MT bundling or spindle stability.

**Figure 3:**
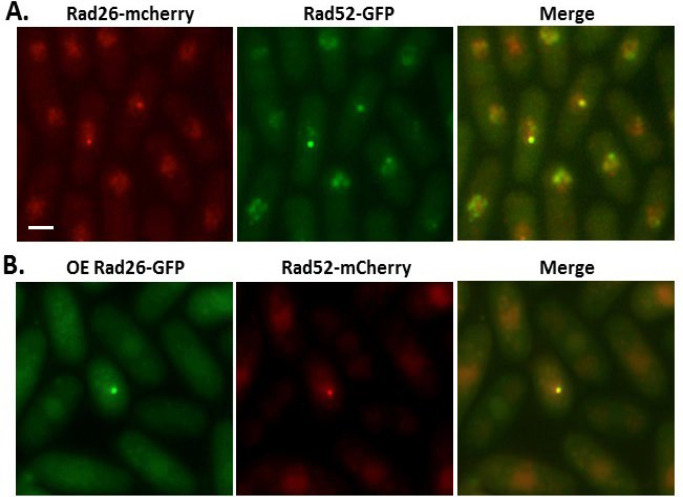
**Rad26 and Rad52 colocalize. A.** Colocalization of Rad26 and Rad52 was observed in cells grown at 30ºC in liquid YE5S to optical density 0.3 and fixed with cold methanol. **B.** Overexpressed Rad26-GFP colocalizes with Rad52-mcherry. Cells were grown in promoter de-repressing liquid conditions (EMM − thiamine) for 20 hours and fixed with methanol. Bar=5 μm

Next, we tested if Rad26^ATRIP^ and Ase1^PRC1^ colocalize with one another. Cycling fission yeast have approximately six faint nuclear Rad26 foci and, occasionally, one or two somewhat brighter foci [32]. We observed that these bright Rad26 foci tangentially localize with Ase1 (Fig. 2E) and MTs (Fig. 2F), but not to the spindle pole body (Fig. 2G). However, we believe that this association between Rad26 and Ase1 is related to the DDR, and not the G_2_-MT damage response, because these bright Rad26 foci co-localized with Rad52 (Fig. 3A). Rad52 is a DNA recombination-promoting protein that displaces RPA-coated ssDNA to facilitate HDR [33]. At sites of DNA damage, Rad52 foci co-localize with DNA checkpoint proteins [34,35] and comigrate with the nuclear envelope-spanning linker of nucleoskeleton and cytoskeleton (LINC) complex [36]. Comigration of Rad52-foci with LINC is a microtubule- and Rad3-dependent process that directs HDR of damaged DNA. Therefore, we suggest that Ase1 tangential localization with Rad26/Rad52-foci is likely playing a role in this microtubule-dependent DDR pathway as opposed to the G_2_-MT damage response. 

**Figure 4:**
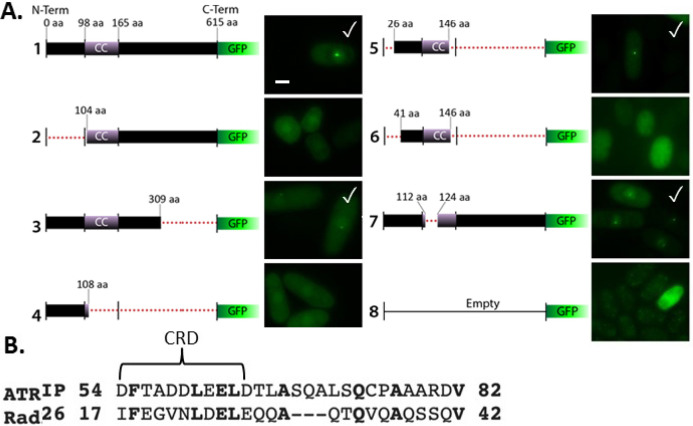
**Rad26 contains an N-terminal Checkpoint Recruitment Domain Required for Focus Formation. A.** Summary of Rad26-GFP truncations. CC = coiled-coil region; ∙∙∙ = DNA removed; ✓ = positive for foci. Bar = 5 μm.** B.** An area that resembles the Checkpoint Recruitment Domain (CRD) of ATRIP [39] lies within the N-terminal region of Rad26 that directs focus formation (25–146aa).

To identify the region of Rad26^ATRIP^ that mediates co-localization with Rad52, we cloned the *rad26*^+^ cDNA in front of GFP in a medium strength thiamine-repressible expression vector. Rad26^ATRIP^ is 615 amino acids long and contains a coiled-coil motif in the N-terminus ([37]; Fig. 4A). Overexpression of the full length Rad26-GFP fusion protein resulted in production of bright nuclear foci that also co-localized with Rad52 (Fig. 3B). A large C-terminal truncation of Rad26^ ATRIP^ did not affect localization to foci (Fig. 4A, construct 3), demonstrating that the N-terminal half of the protein is sufficient for focus formation. A region near the N-terminal border of the coiled-coil was critical for focus formation, since neither 1–108aa (Fig. 4A, construct 4) or 104-615aa (Fig. 4A, construct 2) localized to foci. This led to identification of an N-terminal region within 26-146aa that directed focus formation (Fig. 4A, construct 5). The intact coiled-coil region of Rad26 was not required for focus formation, since an 11 amino acid deletion within the coiled-coil permitted focus formation (Fig. 4A, construct 7). 

Within ATRIP, an N-terminal region between 43–108aa contains a major RPA-ssDNA interacting domain [38]. An acidic *checkpoint recruitment domain *(CRD) exists within this N-terminal region (54–68aa) that mediates ATRIP focus formation and interaction with a basic cleft of RPA subunit RPA70N [39,40]. This CRD is conserved in budding yeast Ddc2^ATRIP^ where it also mediates focus formation and interaction with RPA. While destruction of this motif impairs ATRIP localization to nuclear foci, it does not completely impair ATR signaling. Here, we identify a region resembling the CRD that exists within the 25–146aa region of Rad26 that directed focus formation (Fig. 4A, construct 5; Fig. 4B). Experiments to determine if it is required for Rad3^ATR^ DNA checkpoint signaling in *S. pombe* are planned. 

To our knowledge, this is the first report implicating Ase1^PRC1^ in regulation of the G_2_/M transition. During G_2_, Ase1^PRC1^ bundles and stabilizes interphase MTs [31]. During the transition from G_2_ to M, Ase1^PRC1^ localizes to the duplicated SPBs as they separate, yet Ase1^PRC1^ is not required for this event in wildtype cells [41-43]. Later, Ase1^PRC1^ is required to stabilize the pre-anaphase spindle and then the spindle midzone, where it influences the future site of division [22,23,31,44-47]. Ase1^PRC1^ plays two regulatory roles following entry into mitosis. Prior to metaphase, Cdc2^CDK1 ^phosphorylates target sites in the Ase1-C-term to prevent release of the kinesin-like motor Klp9 from Ase1^PRC1^ [48]. After metaphase, the Clp1/Flp1^CDC14^ phosphatase removes these inhibitory phosphate groups to permit release of Klp9 from Ase1^PRC1^ in order to initiate anaphase B microtubule sliding. Ase1^PRC1^ is also required for the Clp1/Flp1^CDC14^- and septation initiation network (SIN)-dependent cytokinesis checkpoint that inhibits successive nuclear divisions following perturbation of actomyosin ring components [22,49-52]. In summary, Ase1^PRC1^ has well established structural and regulatory roles during later mitotic events, and this report here appears to be the first to identify an earlier G_2_/M role for Ase1^PRC1^.

## Conflict of Interest

We declare there is no conflict of interest to report.

## References

[1] Lieber MR (2010). The mechanism of double-strand DNA break repair by the nonhomologous DNA end-joining pathway. Annu Rev Biochem.

[2] Jasin M, Rothstein R (2013). Repair of strand breaks by homologous recombination. Cold Spring Harb Perspect Biol.

[3] Shiloh Y (2006). The ATM-mediated DNA-damage response: taking shape. Trends Biochem Sci.

[4] Maréchal A, Zou L (2013). DNA damage sensing by the ATM and ATR kinases. Cold Spring Harb Perspect Biol.

[5] Pannunzio NR, Watanabe G, Lieber MR (2018). Nonhomologous DNA end-joining for repair of DNA double-strand breaks. J Biol Chem.

[6] Burger K, Ketley RF, Gullerova M (2019). Beyond the Trinity of ATM, ATR, and DNA-PK: multiple kinases shape the DNA damage response in concert with RNA metabolism. Front Mol Biosci.

[7] Trielli MO, Andreassen PR, Lacroix FB, Margolis RL (1996). Differential Taxol-dependent arrest of transformed and nontransformed cells in the G1 phase of the cell cycle, and specific-related mortality of transformed cells. J Cell Biol.

[8] Giannakakou P, Robey R, Fojo T, Blagosklonny MV (2001). Low concentrations of paclitaxel induce cell type-dependent p53, p21 and G1/G2 arrest instead of mitotic arrest: molecular determinants of paclitaxel-induced cytotoxicity. Oncogene.

[9] Mantel CR, Braun SE, Lee Y, Kim YJ, Broxmeyer HE (2001). The interphase microtubule damage checkpoint defines an S-phase commitment point and does not require p21(waf-1). Blood.

[10] Mantel CR, Gelfano VM, Kim YJ, McDaniel A, Lee Y, Boswell Hs, Broxmeyer HE (2002). P21waf-1-Chk1 pathway monitors G1 phase microtubule integrity and is crucial for restriction transition. Cell Cycle.

[11] Naaz F, Ahmad F, Lone BA, Pokharel YR, Fuloria NK, Fuloria S, Ravichandran M, Pattabhiraman L, Shafi S, Shahar Yar M (2020). Design and synthesis of newer 1,3,4-oxadiazole and 1,2,4-triazole based Topsentin analogues as anti-proliferative agent targeting tubulin. Bioorg Chem.

[12] Cussiol JRR, Soares BL, Oliveira FMB de (2019). From yeast to humans: Understanding the biology of DNA damage response (DDR) kinases. Genet Mol Biol.

[13] Herring M, Davenport N, Stephan K, Campbell S, White R, Kark J, Wolkow TD (2010). Fission yeast Rad26ATRIP delays spindle-pole-body separation following interphase microtubule damage. J Cell Sci.

[14] Balestra FR, Jimenez J (2008). A G2-phase microtubule-damage response in fission yeast. Genetics.

[15] Nurse P (1975). Genetic control of cell size at cell division in yeast. Nature.

[16] Russell P, Nurse P (1987). Negative regulation of mitosis by wee1+, a gene encoding a protein kinase homolog. Cell.

[17] Raleigh JM, O’Connell MJ (2000). The G(2) DNA damage checkpoint targets both Wee1 and Cdc25. J Cell Sci.

[18] Craven RA, Griffiths DJ, Sheldrick KS, Randall RE, Hagan IM, Carr AM (1998). Vectors for the expression of tagged proteins in Schizosaccharomyces pombe. Gene.

[19] Bähler J, Wu JQ, Longtine MS, Shah NG, McKenzie A, Steever AB, Wach A, Philippsen P, Pringle JR (1998). Heterologous modules for efficient and versatile PCR-based gene targeting in Schizosaccharomyces pombe. Yeast.

[20] Bridge AJ, Morphew M, Bartlett R, Hagan IM (1998). The fission yeast SPB component Cut12 links bipolar spindle formation to mitotic control. Genes Dev.

[21] Baschal EE, Chen KJ, Elliott LG, Herring MJ, Verde SC, Wolkow TD (2006). The fission yeast DNA structure checkpoint protein Rad26ATRIP/LCD1/UVSD accumulates in the cytoplasm following microtubule destabilization. BMC Cell Biol.

[22] Yamashita A, Sato M, Fujita A, Yamamoto M, Toda T (2005). The roles of fission yeast Ase1 in mitotic cell division, meiotic nuclear oscillation, and cytokinesis checkpoint signaling. Mol Biol Cell.

[23] Schuyler SC, Liu JY, Pellman D (2003). The molecular function of Ase1p: evidence for a MAP-dependent midzone-specific spindle matrix. Microtubule-associated proteins. J Cell Biol.

[24] Glotzer M (2009). The 3Ms of central spindle assembly: microtubules, motors and MAPs. Nat Rev Mol Cell Biol.

[25] Subramanian R, Wilson-Kubalek EM, Arthur CP, Bick MJ, Campbell EA, Darst SA, Milligan RA, Kapoor TM (2010). Insights into antiparallel microtubule crosslinking by PRC1, a conserved nonmotor microtubule binding protein. Cell.

[26] She ZY, Wei YL, Lin Y, Li YL, Lu MH (2019). Mechanisms of the Ase1/PRC1/MAP65 family in central spindle assembly. Biol Rev Camb Philos Soc.

[27] Fantes P (1979). Epistatic gene interactions in the control of division in fission yeast. Nature.

[28] Hagan IM, Grallert A, Simanis V (2016). Analysis of the Schizosaccharomyces pombe Cell Cycle. Cold Spring Harb Protoc.

[29] Akera T, Sato M, Yamamoto M (2012). Interpolar microtubules are dispensable in fission yeast meiosis II. Nat Commun.

[30] Hartmuth S, Petersen J (2009). Fission yeast Tor1 functions as part of TORC1 to control mitotic entry through the stress MAPK pathway following nutrient stress. J Cell Sci.

[31] Loïodice I, Staub J, Setty TG, Nguyen N-PT, Paoletti A, Tran PT (2005). Ase1p Organizes Antiparallel Microtubule Arrays during Interphase and Mitosis in Fission Yeast. Mol Biol Cell.

[32] Wolkow TD, Enoch T (2003). Fission yeast Rad26 responds to DNA damage independently of Rad3. BMC Genet.

[33] Symington LS (2016). Mechanism and regulation of DNA end resection in eukaryotes. Crit Rev Biochem Mol Biol.

[34] Du LL, Nakamura TM, Moser BA, Russell P (2003). Retention but not recruitment of Crb2 at double-strand breaks requires Rad1 and Rad3 complexes. Mol Cell Biol.

[35] Meister P, Poidevin M, Francesconi S, Tratner I, Zarzov P, Baldacci G (2003). Nuclear factories for signalling and repairing DNA double strand breaks in living fission yeast. Nucleic Acids Res.

[36] Swartz RK, Rodriguez EC, King MC (2014). A role for nuclear envelope-bridging complexes in homology-directed repair. Mol Biol Cell.

[37] De Souza CP, Ye XS, Osmani SA (1999). Checkpoint defects leading to premature mitosis also cause endoreplication of DNA in Aspergillus nidulans. Mol Biol Cell.

[38] Namiki Y, Zou L (2006). ATRIP associates with replication protein A-coated ssDNA through multiple interactions. Proc Natl Acad Sci U S A.

[39] Ball HL, Ehrhardt MR, Mordes DA, Glick GG, Chazin WJ, Cortez D (2007). Function of a conserved checkpoint recruitment domain in ATRIP proteins. Mol Cell Biol.

[40] Xu X, Vaithiyalingam S, Glick GG, Mordes DA, Chazin WJ, Cortez D (2008). The basic cleft of RPA70N binds multiple checkpoint proteins, including RAD9, to regulate ATR signaling. Mol Cell Biol.

[41] Rincon SA, Lamson A, Blackwell R, Syrovatkina V, Fraisier V, Paoletti A, Betterton MD, Tran PT (2017). Kinesin-5-independent mitotic spindle assembly requires the antiparallel microtubule crosslinker Ase1 in fission yeast. Nat Commun.

[42] Yukawa M, Kawakami T, Okazaki M, Kume K, Tang NH, Toda T (2017). A microtubule polymerase cooperates with the kinesin-6 motor and a microtubule cross-linker to promote bipolar spindle assembly in the absence of kinesin-5 and kinesin-14 in fission yeast. Mol Biol Cell.

[43] Ebina H, Ji L, Sato M (2019). CLASP promotes microtubule bundling in metaphase spindle independently of Ase1/PRC1 in fission yeast. Biol Open.

[44] Mollinari C, Kleman J-P, Jiang W, Schoehn G, Hunter T, Margolis RL (2002). PRC1 is a microtubule binding and bundling protein essential to maintain the mitotic spindle midzone. J Cell Biol.

[45] Vernì F, Somma MP, Gunsalus KC, Bonaccorsi S, Belloni G, Goldberg ML, Gatti M (2004). Feo, the Drosophila homolog of PRC1, is required for central-spindle formation and cytokinesis. Curr Biol.

[46] Meadows JC, Millar J (2008). Latrunculin a delays anaphase onset in fission yeast by disrupting an Ase1-independent pathway controlling mitotic spindle stability. Mol Biol Cell.

[47] Expósito-Serrano M, Sánchez-Molina A, Gallardo P, Salas-Pino S, Daga RR (2020). Selective nuclear pore complex removal drives nuclear envelope division in fission yeast. Curr Biol.

[48] Fu C, Ward JJ, Loiodice I, Velve-Casquillas G, Nedelec FJ, Tran PT (2009). Phospho-regulated interaction between kinesin-6 Klp9p and microtubule bundler Ase1p promotes spindle elongation. Dev Cell.

[49] Le Goff X, Woollard A, Simanis V (1999). Analysis of the cps1 gene provides evidence for a septation checkpoint in Schizosaccharomyces pombe. Mol Gen Genet.

[50] Liu J, Wang H, Balasubramanian MK (2000). A checkpoint that monitors cytokinesis in Schizosaccharomyces pombe. J Cell Sci.

[51] Mishra M, Karagiannis J, Trautmann S, Wang H, McCollum D, Balasubramanian MK (2004). The Clp1p/Flp1p phosphatase ensures completion of cytokinesis in response to minor perturbation of the cell division machinery in Schizosaccharomyces pombe. J Cell Sci.

[52] Trautmann S, McCollum D (2005). Distinct nuclear and cytoplasmic functions of the S pombe Cdc14-like phosphatase Clp1p/Flp1p and a role for nuclear shuttling in its regulation. Curr Biol.

